# Long QTc in hypertrophic cardiomyopathy: A consequence of structural myocardial damage or a distinct genetic disease?

**DOI:** 10.3389/fcvm.2023.1112759

**Published:** 2023-04-05

**Authors:** Francesco Cava, Caterina Micolonghi, Maria Beatrice Musumeci, Simona Petrucci, Camilla Savio, Marco Fabiani, Giacomo Tini, Aldo Germani, Fabio Libi, Carla Rossi, Vincenzo Visco, Antonio Pizzuti, Massimo Volpe, Camillo Autore, Speranza Rubattu, Maria Piane

**Affiliations:** ^1^ Department of Clinical and Molecular Medicine, Sapienza University, Rome, Italy; ^2^ Department of Experimental Medicine, Sapienza University of Rome, Rome, Italy; ^3^ Sant'Andrea University Hospital, Rome, Italy; ^4^ Casa Sollievo Della Sofferenza Foundation, San Giovanni Rotondo, Italy; ^5^ IRCCS S.Raffaele, Rome, Italy; ^6^ IRCCS S.Raffaele, Cassino, Italy; ^7^ IRCCS Neuromed, Pozzilli, Italy

**Keywords:** hypertrophic cardiomyopathy, QTc, LQTS, gene muatation, QTc abnormalities

## Abstract

Hypertrophic cardiomyopathy (HCM) is an autosomal dominant disease, characterized by the presence of unexplained left ventricular hypertrophy. This condition is often associated with electrocardiographic abnormalities including QTc prolongation occurring in 13% of patients. The main explanation for prolonged QTc in HCM is myocardial hypertrophy and the related structural damage. However, other mechanisms, including long QT syndrome (LQTS) genes mutations, may be involved. In the present study we explored the hypothesis of a distinct genetic basis underlying QTc prolongation in HCM by investigating the potential co-inheritance of pathogenic gene variants associated with LQTS and HCM. For this purpose, starting from a cohort of 150 HCM patients carrying pathogenic variants in sarcomere genes, we selected 25 patients carrying a QTc prolongation unexplained by any other cause. The QTc was considered prolonged if greater than 450 ms in males and greater than 470 ms in females. The NGS analysis was performed with Illumina TrueSight Cardio panel genes on Illumina MiniSeq platform. We identified pathogenic/likely pathogenic variants in the *KCNQ1* in two patients (c.1781G > A, p. Arg594Gln; c.532G > A, p. Ala178Thr) (8%). Variants of uncertain significance were identified in *SCN5A*, *KCNJ5*, *AKAP9* and *ANK2* in four patients (16%). Although the results are limited by the small number of patients included in the study, they highlight a minor contribution of LQTS genes for QTc prolongation in HCM patients. The screening for ion channel genes mutations may be considered in HCM patients with prolonged QTc unexplained by any other cause. This in-depth molecular diagnosis may contribute to improve risk stratification and treatment planning.

## Introduction

Hypertrophic cardiomyopathy (HCM) is a condition characterized by the presence of left ventricular asymmetric hypertrophy and is one of the most common cardiovascular genetic diseases, inherited with autosomal pattern and incomplete penetrance (1). This condition is often associated with electrocardiographic abnormalities, including QTc prolongation occurring in 13% of HCM patients (2). The main explanation for QTc prolongation in HCM appears to be myocardial hypertrophy and the related structural damage. In fact, the hypertrophy distribution and magnitude, the myocardial disarray, the subendocardial ischemia as well as the myocardial fibrosis areas, all typical features of HCM, worsen the transmural dispersion of myocardial repolarization and, contextually, increase the arrhythmic risk (3). However, it has been observed that patients with HCM-related genes mutations frequently exhibit impaired QT variables even in the absence of ventricular hypertrophy (4, 5).

To explain the prolonged QTc in HCM, the role of gene mutations involved in the LQTS syndrome cannot be excluded. In fact, the additive effect of 2 disease-causing mutations has been reported in other pathological conditions (6). Moreover, recent evidence shows that a comprehensive genetic testing for cardiomyopathies and arrhythmias reveals genes variants that would have been missed by disease-specific testing (7).

In the present study we investigated the possible co-inheritance of pathogenic variants associated with LQTS and HCM, exploring the hypothesis of a distinct genetic basis underlying QTc prolongation in HCM.

## Materials and methods

For this study we considered a prospectively collected database, including 150 consecutive HCM outpatients carrying pathogenic variants in sarcomere genes, based on previous genetic screenings, referred to the tertiary HCM Center of Sant’Andrea Hospital, Sapienza University of Rome between January 2014 and June 2021.

Each patient was evaluated considering the HCM echocardiographic findings detected by transthoracic Doppler echocardiography (apical, septal hypertrophy), the electrocardiographic findings, the clinical history and New York Heart Association (NYHA) classification, the pedigree analysis. The following echocardiographic parameters were considered: Left Ventricular (LV) end-diastolic diameter (LVEDd, parasternal long axis), LV maximal wall thickness (MWT, measured at any LV site), left atrial diameter (LAd, parasternal long axis), the highest maximal LV outflow tract gradient among those measured at rest, in the orthostatic position and after the Valsalva maneuver (LVOTGmax, apical 4-chamber view), LV ejection fraction assessed with Simpson's biplane methods (LVEF, apical 4-chamber view). The diagnosis of HCM was based on a MWT of 15 mm unexplained by abnormal loading conditions or in accordance with published criteria for the diagnosis of disease in relatives of patients with unequivocal disease (8).

The QTc was considered prolonged if greater than 450 ms in males and greater than 470 ms in females and it was unexplained by any other cause (9). Exclusion criteria were the following: ECG evidence of complete left/right bundle branch block, pacemaker-dependent rhythm, or other arrhythmias likely to interfere with assessments, frequent extrasystoles. Finally, patients taking any antiarrhythmic drugs (except for beta-blockers) at the study evaluation, as well as those patients with metabolic diseases or syndromic causes of HCM (patients affected by Fabry Disease, Noonan syndrome, and with Leopard syndrome) were excluded from the study.

Accordingly, a total of 25 HCM patients carrying pathogenic variants in sarcomere genes and QTc prolongation not explained by any other cause was selected for the study purpose.

The study complied with the ethical standards of the Declaration of Helsinki and was reviewed and approved by the institutional ethics committee of our center. Written informed consent was obtained from all participants.

### 12-lead surface ECG data analysis

After a 5-min rest in the supine position, each HCM patient underwent 12-lead surface ECG at 25 mm/s. The latter was used to manually measure each interval length in every lead with a digital caliper (Cardio Caliper, Iconico, New York, NY, USA). The following intervals were obtained: R-R, Q-R-S, Q-Tend (QT, from the Q wave to the T wave end), Q-T peak (QTp, from the Q wave to the T wave peak), Tpeak-Tend (TpTe, difference between QT and QTp), J-Tend (JT, difference between QT and QRS), J-Tpeak (JTp, difference between QTp and QRS). The QT interval was measured from the onset of the QRS complex to the end of the T wave. To minimize the possible confounder of different heart rate values, each interval was also corrected according to Bazett's classic formula. In patients with more than one ECG with QTcBazett ≥ 450 ms during the study period, only the first ECG was chosen.

### Next generation sequency (NGS) analysis

NGS analysis was performed with Illumina TrueSight Cardio panel genes on Illumina MiniSeq Platform (Life Technology) to evaluate the presence of pathogenetic variants in LQTS related genes. Genomic DNA included was extracted from peripheral whole blood of the patients and used for the preparation of libraries with the TruSight Cardio Panel Kit (Illumina) and subsequently analyzed on the Illumina MiniSeq Sequencer. The TruSight Cardio Panel kit exploiting the NGS methodology provides complete coverage of 174 genes associated with known hereditary heart diseases, including 14 related to monogenic LQT syndromes (*AKAP9, ANK2, CACNA1C, CAV3, KCNE1, KCNE2, KCNH2, KCNJ2, KCNJ5, KCNQ1, SCN4B, SCN5A, SNTA1, TRDN*). The known variants identified were classified according to the American College of Medical Genetics and Genomics criteria (10). New variants found were analyzed using three types of prediction software (SIFT, POLYPHEN and PROVEAN) and classified according to both their agreement and the presence of studies in the literature. The pathogenetic variants identified were confirmed with Sanger sequencing using the standard protocol with specific primers expressly.

## Results

The clinical, echocardiographic, and electrocardiographic characteristics of the 25 patients included in the analysis, along with the previously identified sarcomere gene mutations, are reported in Supplementary Tables S1, S2. Most of the patients included in the study showed septal hypertrophy at the echocardiographic examination. They did not show any marked alterations of ventricular repolarization at the electrocardiogram because of left ventricular hypertrophy. Many of the patients were symptomatic for dyspnea (NYHA II-III) whereas only a minority of them has syncopal episodes. They did not undergo MRI.

We identified pathogenic/likely pathogenic variants in the LQTS related gene *KCNQ1* (c.1781G > A, p. Arg594Gln; c.532G > A, p. Ala178Thr) in two patients (8%). Moreover, few uncertain significant variants were identified in *SCN5A*, *KCNJ5*, *AKAP9* and *ANK2* in four patients (16%).

## Discussion

The presence of long QTc is found in about 13% of HCM patients (11) and is considered a predictor for the need of ICD (Implantable Cardioverter-Defibrillator) implantation. As previously reported, the QTc length is a predictor of sudden death in HCM (1, 12). Concerning the cause of QTc abnormalities in HCM, either a structural or a genetic origin can be hypothesized. The first hypothesis suggests that QTc abnormalities derive mainly from the repolarization dispersion due to the myocardial hypertrophy. The second hypothesis proposes the independent genetic origin of the prolonged QTc length in HCM. In this regard, an early study reported the impact of polymorphisms in the LQT related gene *NOS1AP* on QTc interval duration (13). More recently, the existence of a Chinese family carrying pathogenic variants related to both HCM and LQTS phenotypes has been reported (14). Moreover, our group recently described a single case of a 24-year-old male, affected by HCM and long QTc, carrying pathogenic variants in both *TNNI3* and *KCNQ1* genes (15). Another case-report underscored the association of a *de novo CALM2* mutation with LQTS and HCM (16). Therefore, the hypothesis of a genetic origin of QTc prolongation in HCM has received some support from the existing literature.

In the present study, performed in highly selected HCM patients to further explore the existence of a distinct genetic origin of prolonged QTc, we identified pathogenic/likely pathogenic variants in the LQTS related gene *KCNQ1* in two patients. We also identified uncertain significant variants in *SCN5A*, *KCNJ5*, *AKAP9* and *ANK2* in four patients. Therefore, the results of our study highlight a minor contribution of LQTS genes in HCM patients carrying prolonged QTc, and they suggest that other mechanisms should be taken into consideration to explain the electrocardiographic abnormality of HCM patients.

Cardiac myocytes are well coupled electrically by gap junctions in normal hearts, resulting in a rapid propagation of the electrical impulse and repolarization. Experimental models reveal that cardiac pathologies induce heterogeneous changes in myocyte electrophysiology, which primarily affect the repolarization phase of the action potential, leading to increased transmural dispersion of ventricular repolarization (17).

The lengthening of the QT interval is mostly linked to mechanical overload and to the ensuing compensatory hypertrophy. Notably, the acquired lengthening of the QT interval is not associated with cardiac hypertrophy of any other origin than mechanical overload, such as cardiac hypertrophy due to thyrotoxicosis. By contrast, the inherited long-QT, and the drug- or metabolic-induced acquired long-QT are not merely associated with cardiac hypertrophy.

The possible mechanisms underlying increased QT dispersion in HCM are ion channels abnormalities (18) and gap junction remodeling (19), leading to increased action potential duration, decreased conduction velocity, and increased spatiotemporal dispersion of repolarization (20), which would manifest as QRS prolongation and increased QT dispersion on surface electrocardiogram. An important feature of HCM is the lack of homogeneity of the distribution of left ventricular hypertrophy, which has been shown to predispose to increased QT dispersion (21). Moreover, the associated fibrosis represents another possible contributor to increased QT dispersion since it could slow impulse propagation and enhance dispersion of repolarization (22).

## Study limitation

The small number of patients enrolled in the study, selected at one single tertiary HCM center, represents an obvious limitation. Further studies in larger HCM cohorts are warranted to reinforce the current evidence.

## Conclusions

Although the results of our study are limited by the small number of patients included in the analysis, they highlight a minor contribution of LQTS genes mutations in HCM patients carrying a QTc prolongation and they suggest that other causes could be involved. On the other hand, it is known that patients with HCM-related genes mutations may exhibit impaired QT variables even in the absence of ventricular hypertrophy (23, 24).

Based on the results of the present analysis, and consistently with recent evidence obtained through a comprehensive genetic testing for cardiomyopathies and arrhythmias (7), we suggest that the screening for ion channel gene mutations may be considered in those HCM patients with a QTc prolongation unexplained by any other cause. This in-depth molecular diagnostic strategy may help improving risk stratification and treatment planning.

## Data Availability

The datasets presented in this study can be found in online repositories. The names of the repository/repositories and accession number(s) can be found in the article/**Supplementary Material**.
