# Supplementary material for: Long QTc in hypertrophic cardiomyopathy: A consequence of structural myocardial damage or a distinct genetic disease?
Source: Front Cardiovasc Med. 2023 Apr 5;10:1112759. doi: 10.3389/fcvm.2023.1112759 (PMC10113437; doi:10.3389/fcvm.2023.1112759)
Supplement: Supplementary file 1 [file Table1.docx]

| ***Patient*** | ***Age*** | ***Sex*** | ***Family history of HCM*** | ***Family history of sudden death*** | ***ICD*** | ***Distribution of Left ventricular hypertrophy*** | ***Maximall wall thickness*** | ***Outflow obstruction*** | ***NSTV/VT/OHCA*** | ***NYHA (I-IV)*** | ***QTc*** |
| --- | --- | --- | --- | --- | --- | --- | --- | --- | --- | --- | --- |
| 1 | 52 | M |  |  |  | septal | 28 mm |  |  | II-III | 467 |
| 2 | 76 | F | Yes | Yes | Yes | septal | 27 mm | Yes |  | III | 510 |
| 3 | 52 | F |  |  |  | septal | 19 mm | Yes |  | III | 463 |
| 4 | 67 | M | Yes |  |  | septal | 28 mm | Yes |  | II-III | 453 |
| 5 | 18 | M | Yes |  | Yes | apical | 31 mm |  |  | II | 453 |
| 6 | 74 | M | Yes | Yes |  | septal | 28 mm | Yes |  | II-III | 473 |
| 7 | 73 | M |  |  |  | apical | 22 mm |  | Yes | I-II | 463 |
| 8 | 54 | F |  |  |  | septal | 17 mm |  |  | II | 464 |
| 9 | 51 | F |  | Yes |  | septal | 21 mm |  |  | II | 461 |
| 10 | 43 | M | Yes |  |  | apical | 25 mm | Yes |  | II | 515 |
| 11 | 60 | F | Yes |  |  | septal | 24 mm |  |  | II-III | 461 |
| 12 | 54 | F |  |  |  | septal | 19 mm |  |  | II | 566 |
| 13 | 67 | F | Yes | Yes | Yes | septal | 21 mm |  | Yes | II-III | 497 |
| 14 | 39 | M |  |  |  | septal | 16 mm |  |  | I-II | 455 |
| 15 | 70 | M |  |  | Yes | concentric | 23 mm |  |  | III-IV | 456 |
| 16 | 47 | M |  |  |  | apical | 18 mm |  |  | II-III | 460 |
| 17 | 55 | F |  |  | Yes | septal | 21 mm |  | Yes | I-II | 468 |
| 18 | 58 | M |  |  | Yes | apical | 30 mm | Yes | Yes | III-IV | 481 |
| 19 | 47 | M |  |  |  | basal anterior | 24 mm |  |  | II | 471 |
| 20 | 33 | M | Yes | Yes |  | septal | 35 mm | Yes |  | II-III | 470 |
| 21 | 41 | F | Yes | Yes | Yes | biventricular | 27 mm |  |  | III | 467 |
| 22 | 51 | M | Yes |  | Yes | septal | 30 mm | Yes | Yes | III-IV | 516 |
| 23 | 69 | M |  |  |  | septal/apical | 27 mm |  |  | II | 461 |
| 24 | 63 | M | Yes |  |  | septal | 23 mm | Yes |  | II-III | 486 |
| 25 | 23 | M | Yes |  |  | septal | 26 mm |  |  | I-II | 480 |

**Table 1**

Legend to Table 1: The table contains all clinical, echocardiographic, cardiac functional parameters and the QTc length of the 25 patients included in the study

*Abbreviations****: HCM***: Hypertrophyc Cardiomiopaty; ***ICD***: Implantable Cardioverter Defibrillator; ***NSVT***: Non-sustained Ventricular Tachycardia; ***VT***: Ventricular Tachycardia; ***OHCA***: Out of Hospital Cardiac Arrest; ***NYHA***: New York Heart Association
