# Supplementary material for: Long QTc in hypertrophic cardiomyopathy: A consequence of structural myocardial damage or a distinct genetic disease?
Source: Front Cardiovasc Med. 2023 Apr 5;10:1112759. doi: 10.3389/fcvm.2023.1112759 (PMC10113437; doi:10.3389/fcvm.2023.1112759)
Supplement: Supplementary file 2 [file Table2.docx]

| **Patient** | **Gene** | **HCM mutation** | **Gene** | **HCM mutation** | **Gene** | **Long QT mutation** | **Gene** | **Long QT mutation** |
| --- | --- | --- | --- | --- | --- | --- | --- | --- |
| **1** | ***MYBPC3*** | c.3192dup, p.Lys1065Glnfs*12 (rs397516007) |  |  |  |  |  |  |
| **2** | ***TNNI3*** | c.549G>T, p.Lys183Asn |  |  |  |  |  |  |
| **3** | ***MYBPC3*** | c.2654C>T, p.Thr885Met (rs397515981) |  |  |  |  |  |  |
| **4** | ***MYH7*** | c.2539_2541del, p.Lys847del ([rs397516155](http://www.ncbi.nlm.nih.gov/projects/SNP/snp_ref.cgi?rs=rs397516155)) |  |  |  |  |  |  |
| **5** | ***MYBPC3*** | c.1505G>A; p.Arg502Glu ( rs397515907) |  |  |  |  |  |  |
| **6** | ***TNNI3*** | c.-8G>A (rs773513015 ) |  |  |  |  |  |  |
| **7** | ***MYBPC3*** | c.2309-2A>G (rs111729952 ) |  |  | ***SCN5A*** | c.2548G>A, p.Val850Met ([rs911293694](http://www.ncbi.nlm.nih.gov/projects/SNP/snp_ref.cgi?rs=rs911293694)) |  |  |
| **8** | ***MYH7*** | c.2167C>T, p. Arg723Cys(rs121913630) |  |  | ***KCNJ5*** | c.968T>C, p.Met323Thr ([rs886048011](http://www.ncbi.nlm.nih.gov/projects/SNP/snp_ref.cgi?rs=rs886048011)) |  |  |
| **9** | ***MYBPC3*** | c.1505G>A; p.Arg502Glu (rs397515907 ) |  |  | ***AKAP9*** | c.10718G>A, p.Ser3573Asn |  |  |
| **10** | ***TNNT2*** | c.832C>T, p.Arg278Cys (rs121964857 ) |  |  | ***KCNQ1*** | c.532G>A, Ala178Thr ([rs120074177](http://www.ncbi.nlm.nih.gov/projects/SNP/snp_ref.cgi?rs=rs120074177)) | ***ANK2*** | c.7648G>A, p.Val2550Ile ([rs1041196848](http://www.ncbi.nlm.nih.gov/projects/SNP/snp_ref.cgi?rs=rs1041196848)) |
| **11** | ***MYBPC3*** | c.787G>A, p.Gly263Arg (rs373730381 ) | ***TNNT2*** | c.330T>G, p.Phe110Leu (rs727504331) |  |  |  |  |
| **12** | ***TNNI3*** | c.592C>G, p.Leu198Val(rs727504285 ) | ***SCN5A*** | c.2077C>T, p.Arg693Cys (rs375306544) |  |  |  |  |
| **13** | ***TNNI3*** | c.592C>G, p.Leu198Val (rs727504285 ) |  |  | ***KCNJ5*** | c.968T>C, p.Met323Thr ([rs886048011](http://www.ncbi.nlm.nih.gov/projects/SNP/snp_ref.cgi?rs=rs886048011)) | ***AKAP9*** | c.1297C>G, p.Leu433Val |
| **14** | ***TNNT2*** | c.418C>T, p.Arg140Cys(rs397516463 ) |  |  |  |  |  |  |
| **15** | ***MYBPC3*** | c.927-2A>G (rs397516082 ) | ***CSRP3*** | c.298C>T, p.Arg100Cys (rs201214593) |  |  |  |  |
| **16** | ***TNNC1*** | c.430A>G, p.Asn144Asp(rs730881061) |  |  |  |  |  |  |
| **17** | ***MYH7*** | c.746G>A; p.Arg249Gln (rs3218713 ) |  |  |  |  |  |  |
| **18** | ***TNNT2*** | c.832C>T, p.Arg278Cys(rs121964857 ) |  |  |  |  |  |  |
| **19** | ***SCN5A*** | c.5239G>A, p.Val174Met (rs199473630) |  |  |  |  |  |  |
| **20** | ***MYBPC3*** | c.966G>A, p.Trp322* (rs727503211) |  |  |  |  |  |  |
| **21** | ***TNNI3*** | c.566G>A, p.Gly189Glu ( rs587782980) |  |  |  |  |  |  |
| **22** | ***MYH7*** | c.2795T>A, p.Met932Lys |  |  |  |  |  |  |
| **23** | ***MYBPC3*** | c.2309-2A>G(rs111729952) |  |  |  |  |  |  |
| **24** | ***MYBPC3*** | c.3192_3192dup, p.Lys1065fs (rs397516007) |  |  |  |  |  |  |
| **25** | ***TNNI3*** | c.592C>G, p.Leu198Val ( [rs727504285](http://www.ncbi.nlm.nih.gov/projects/SNP/snp_ref.cgi?rs=rs727504285)) |  |  | ***KCNQ1*** | c.1781G>A, p.Arg594Gln ([rs199472815](http://www.ncbi.nlm.nih.gov/projects/SNP/snp_ref.cgi?rs=rs199472815)) |  |  |

**Table 2**

Legend to Table 2

The table contains the description of the sarcomere genes mutations and of the Long QT genes mutations, when present, in the 25 patients included in the study.

Abbreviations: ***MYBPC3****, myosin binding protein C3;* ***TNNI3****, troponin I 3, cardiac type;* ***MYH7****, myosin heavy chain 7;* ***TNNT2****, troponin T2 cardiac type;* ***SCN5A****,* Sodium Voltage-Gated Channel Alpha Subunit 5; ***KCNJ5****,* potassium inwardly rectifying channel subfamily J member 5; ***AKAP9****,* A-Kinase Anchoring Protein 9; ***KCNQ1****,* potassium voltage-gated channel subfamily Q member 1; ***ANK2****,* Ankyrin 2; **CSRP3**, Cysteine and Glycine Rich Protein 3)
